# The Contribution of Mast Cells to the Regulation of Elastic Fiber Tensometry in the Skin Dermis of Children with Marfan Syndrome

**DOI:** 10.3390/ijms25179191

**Published:** 2024-08-24

**Authors:** Dmitrii Atiakshin, Ekaterina Nikolaeva, Alla Semyachkina, Andrey Kostin, Artem Volodkin, Sergey Morozov, Michael Ignatyuk, Liudmila Mikhaleva, Grigory Demyashkin, Daniel Elieh-Ali-Komi, Igor Buchwalow, Markus Tiemann

**Affiliations:** 1 RUDN University, 6 Miklukho-Maklaya St., 117198 Moscow, Russian Federation; andocrey@mail.ru (A.K.); volodkin-av@rudn.ru (A.V.); ignatyuk-ma@rudn.ru (M.I.); buchwalow@pathologie-hh.de (I.B.); 2 Research Institute of Experimental Biology and Medicine, Burdenko Voronezh State Medical University, 394036 Voronezh, Russia; 3 Veltischev Research and Clinical Institute for Pediatrics & Pediatric Surgery of the Pirogov Russian National Research Medical University, 2, Taldomskaya St., 125412 Moscow, Russia; kate_nikolaeva09@mail.ru (E.N.); asemyachkina@pedklin.ru (A.S.); mser@list.ru (S.M.); 4 Avtsyn Research Institute of Human Morphology, Petrovsky National Research Center of Surgery, 119435 Moscow, Russia; mikhalevalm@yandex.ru; 5 Laboratory of Histology and Immunohistochemistry, I.M. Sechenov First Moscow State Medical University (Sechenov University), Trubetskaya St., 8/2, 119048 Moscow, Russia; dr.dga@mail.ru; 6 Institute of Allergology, Charité—Universitätsmedizin Berlin, Corporate Member of Freie Universität Berlin and Humboldt-Universität zu Berlin, 10117 Berlin, Germany; daniel.elieh-ali-komi@charite.de; 7 Allergology and Immunology, Fraunhofer Institute for Translational Medicine and Pharmacology ITMP, 12203 Berlin, Germany; 8 Institute for Hematopathology, Fangdieckstr, 75a, 22547 Hamburg, Germany; mtiemann@hp-hamburg.de

**Keywords:** Marfan syndrome, mast cells, skin, tryptase, chymase, carboxypeptidase A3, heparin, elastic fibers, tensometry extracellular matrix remodeling

## Abstract

Marfan syndrome (MFS) is a hereditary condition accompanied by disorders in the structural and regulatory properties of connective tissue, including elastic fibers, due to a mutation in the gene encodes for fibrillin-1 protein (FBN1 gene) and the synthesis of abnormal fibrillin-1 glycoprotein. Despite the high potential of mast cells (MCs) to remodel the extracellular matrix (ECM), their pathogenetic significance in MFS has not been considered yet. The group of patients with Marfan syndrome included two mothers and five children (three girls aged 4, 11, and 11 and two boys aged 12 and 13). Normal skin was examined in two children aged 11 and 12. Histochemical, monoplex, and multiplex immunohistochemical techniques; combined protocols of simultaneous histochemical and immunohistochemical staining (the results of staining were assessed using light, epifluorescence, and confocal microscopy); and bioinformatics algorithms for the quantitative analysis of detected targets were used to evaluate mast cells and their relationship with other cells from extracellular structures in the skin dermis. Analysis of the skin MC population in children with Marfan syndrome revealed a considerably increased number of intra-organic populations with the preservation of the specific Tryptase^+^Chymase^+^CPA3^+^ protease profile typical of the skin. The features of the MC histotopography phenotype in MFS consisted of closer colocalization with elastic fibers, smooth muscle cells, and fibroblasts. MCs formed many intradermal clusters that synchronized the activity of cell functions in the stromal landscape of the tissue microenvironment with the help of spatial architectonics, including the formation of cell chains and the creation of fibrous niches. In MCs, the expression of specific proteases, TGF-β, and heparin increased, with targeted secretion of biologically active substances relative to the dermal elastic fibers, which had specific structural features in MFS, including abnormal variability in thickness along their entire length, alternating thickened and thinned areas, and uneven surface topography. This paper discusses the potential role of MCs in strain analysis (tensometry) of the tissue microenvironment in MFS. Thus, the quantitative and qualitative rearrangements of the skin MC population in MFS are aimed at altering the stromal landscape of the connective tissue. The results obtained should be taken into account when managing clinical signs of MFS manifested in other pathogenetically critical structures of internal organs, including the aorta, tendons, cartilage, and parenchymal organs.

## 1. Introduction 

Marfan syndrome (MFS), described a little over 100 years ago, is one of the first conditions classified as an inherited connective tissue disorder. The prevalence of MFS is 1 per 3000–5000 people in the overall population, with an autosomal dominant type of inheritance [1]. MFS is based on mutations in the gene encodes for the fibrillin-1 glycoprotein–*FBN1* gene (15q21.1). Fibrillin-1 is localized in the extracellular matrix (ECM) of the connective tissue, forming microfibrils, and it is an essential element in the processing and physiology of elastic fibers. Fibrillin-1 monomers form a structure that helps generate mature elastic fibers from tropoelastin [2]. In these circumstances, microfibrils act as a scaffold for elastin and support the assembly process with the formation of a functional architecture of elastic fibers [3,4]. Elastic fibers provide the ECM with plasticity and resistance to stress in the skin, blood vessel membranes, ligaments, tendons, cartilage, the “soft skeletons” of parenchymal organs, and other structures. Differences found in the skin of most patients with MFS are primarily related to a decreased number of fibrillin-1 microfibrils. In the fibroblasts of children with neonatal MFS, microfibril shortening and fragmentation have been detected [5]. Research investigating fibroblasts has provided findings on decreased synthesis, secretion, and integration of fibrillin-1 into the ECM in MFS [6]. Simulations of the condition in laboratory rodents have demonstrated a decreased density of elastic fibers and the disorganization of microfibrils in the cornea of mice. In addition to its structural relevance, fibrillin-1 has a regulatory function, affecting the storage, release, and activation of transforming growth factor-beta (TGF-β), which, in turn, exhibits a wide range of biological effects [7,8,9].

Mast cells (MCs) are part of innate immunity, and their involvement in the initiation and pathogenesis of allergic diseases is well documented in numerous studies [10,11,12]. However, the physiological and pathological implications of MCs extend far beyond the realization of both innate and specific immunity [13,14,15]. As it is known, MCs are crucial in fibrosis and ECM remodeling [16,17,18], indicating the involvement of connective tissue homeostasis in the pathogenesis under MFS. MCs participate in the creation of profibrogenic tissue niches, in which favorable conditions for the development of fibrotic changes are formed. The presence of specific proteases in mast cells—tryptase, chymase, and carboxypeptidase A3—and other components of the secretome with high biological activity, including TGF-β, allows providing conditions for the disassembly of fibrous structures and fibrillogenesis to process new fibrous elements adequately relative to existing conditions [18]. Notably, the processes of fiber degradation and neoformation with MC participation can be implemented simultaneously, giving MCs the sculptor role of a specific tissue microenvironment [14,18]. The synthesis of a large number of cytokines and growth factors enables MCs to actively influence the collagen- and elastin-producing elements of connective tissue, adapting the composition of the ECM to function under certain environmental conditions [19,20]. This role of MCs is clearly manifested in wound regeneration at each of the healing phases, including inflammation, proliferation, and remodeling. MC activation, with the release of pre-formed or de novo synthesized mediators, including histamine, specific proteases, VEGF, IL-6, IL-8, bFGF, NGF, and PDGF, results in alterations in capillary permeability, vasodilation, and directed migration of granulocytes, lymphocytes, monocytes, and macrophages. It also stimulates the division and increased biosynthetic activity of fibroblasts, results in an increased number of myofibroblasts, enhances collagen synthesis, and promotes neoangiogenesis [14]. Thus, the fact that MCs are involved in the pathogenesis of MFS at the level of the stromal landscape of a specific tissue microenvironment can be promising in terms of pharmacological targets. Moreover, in MFS, abnormal microfibrils are to some extent deprived of the function of structural tissue “tensometers”, modifying the ability of both stromal and immunocompetent cells of the dermis to adequately monitor the state of the integrative buffer metabolic environment, including responses to mechanical and gravitational exposure. Since MCs have been found in close contact with elastic fibers under a number of pathophysiological conditions [21,22], and they synthesize enzymes capable of causing elastic fiber remodeling, including elastase and cathepsin G [23,24,25,26,27,28], we could assume their specific biological features in Marfan syndrome. Since we could not find research investigating MCs under this hereditary condition, the aim of our study is not only to investigate the features of the MC population in Marfan syndrome for the first time but also to evaluate the potential role of MCs in the tensometry of the local tissue microenvironment in the skin dermis as one of the essential functions of elastic fibers.

## 2. Results

### 2.1. Normal Skin

**Figure 1:**
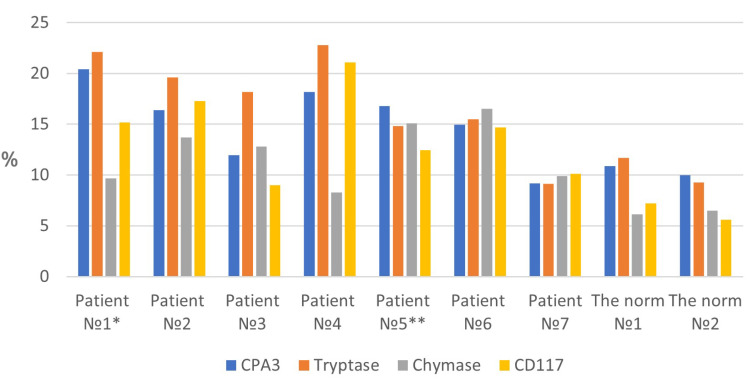
Mast cell content in the skin of patients with Marfan syndrome (in %, relative content to other cells in the dermis). Notes: *—mother of patient No. 2; **—mother of patients No. 6 and No. 7; CPA3—Carboxypeptidase A3.

The relative number of MCs with a high content of tryptase and CPA3 was about 10% of all cells in the skin dermis, while Chymase^high^ MCs and CD117^+^ MCs were lower (Figure 1, Supplementary Table S1). In some MCs with the Tryptase^high^ and CPA3^high^ phenotype, chymase content was low, creating a certain balance to enhance expression if necessary. These data were consistent with the results of multiplex immunohistochemical detection of specific proteases, according to which almost all skin MCs had the Tryptase^+^Chymase^+^CPA3^+^ phenotype, which is typical of the connective tissue type of MCs.

**Figure 2:**
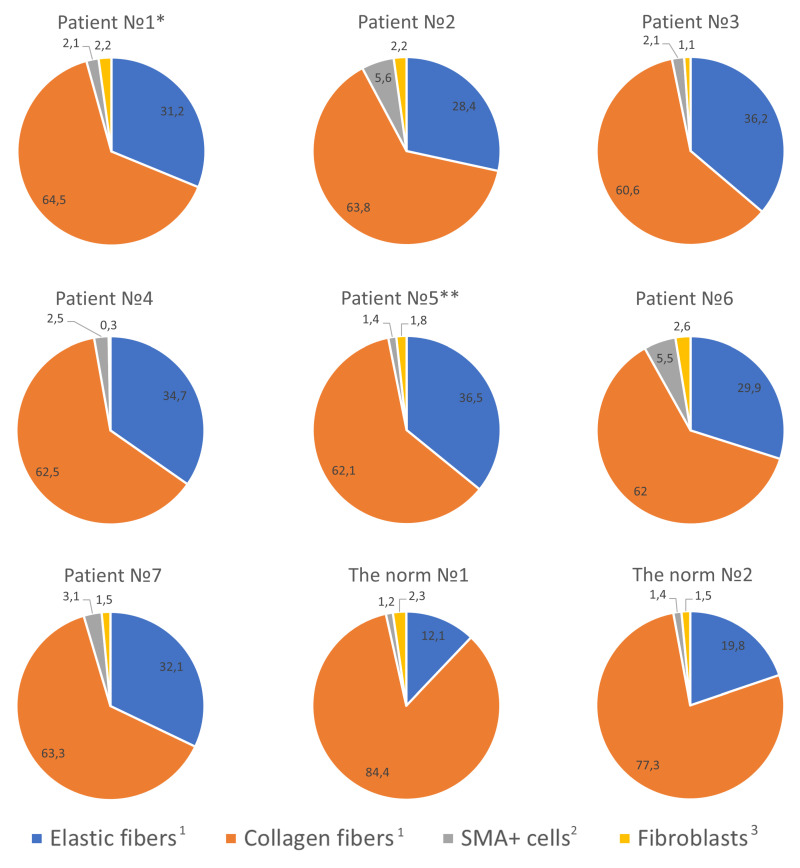
Histotopography of tryptase-positive mast cells in the skin dermis of patients with Marfan syndrome. Frequency of colocalization of MCs (%) with a fibrous component, αSMA-positive cells, and fibroblast. Staining technique: ^1^ multiplex detection of tryptase, elastic, and collagen fibers; ^2^ multiplex immunohistochemical detection of tryptase and α-SMA; ^3^ Giemsa staining. α-SMA: alpha-smooth muscle actin. Notes: *—mother of patient No. 2; **—mother of patients No. 6 and No. 7.

MC colocalization with collagen and elastic fibers seemed interesting. Frequently, adjacency to the fibrous component of the extracellular matrix was observed, and active fiber-oriented secretion was detected as well. As a rule, thicker elastic and collagen fibers were observed in the reticular layer of the dermis, while thinner ones were observed in the papillary layer. In general, MCs were more frequently in contact with collagen fibers than with elastic fibers (Figure 2, Supplementary Table S2). MC colocalization with SMA-positive cells was rare, and this was most often detected when MCs were adjacent to the vessels in the skin dermis (Figure 2, Supplementary Table S2).

**Figure 3:**
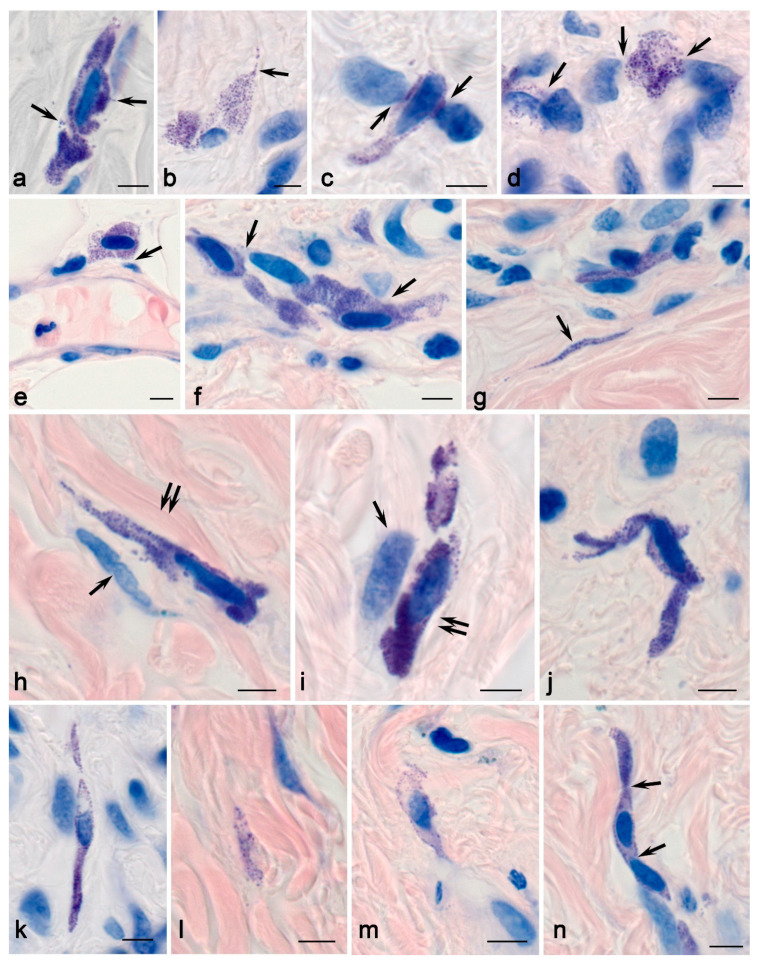
Skin mast cells in children without pathology. Technique: Giemsa staining. (**a**) Two contacting MCs with signs of secretion (arrowed). (**b**) MCs in the skin dermis: one of the cells has a long, narrow process (arrowed). (**c**,**d**) MC colocalization with fibroblasts (arrowed). (**e**) Adjacency of an MC to the postcapillary wall (arrowed). (**f**) A group of MCs in contact with each other and fibroblasts (arrowed). (**g**) MCs and a nuclear-free MC fragment located between bundles of collagen fibers (arrowed). (**h**,**i**) Elongated MCs in contact with fibrocytes (presumably arrowed) and collagen fibers (double arrowed). (**j**) MC that forms an outgrowth in the skin dermis. (**k**) An elongated MC adjacent to two fibroblasts. (**l**) A nuclear-free MC fragment among bundles of collagen fibers. (**m**) A degranulated MC with a predominantly peripheral localization of secretory granules. (**n**) MCs in contact with each other (arrowed), forming a functional chain throughout. Scale: 5 µm.

**Figure 4:**
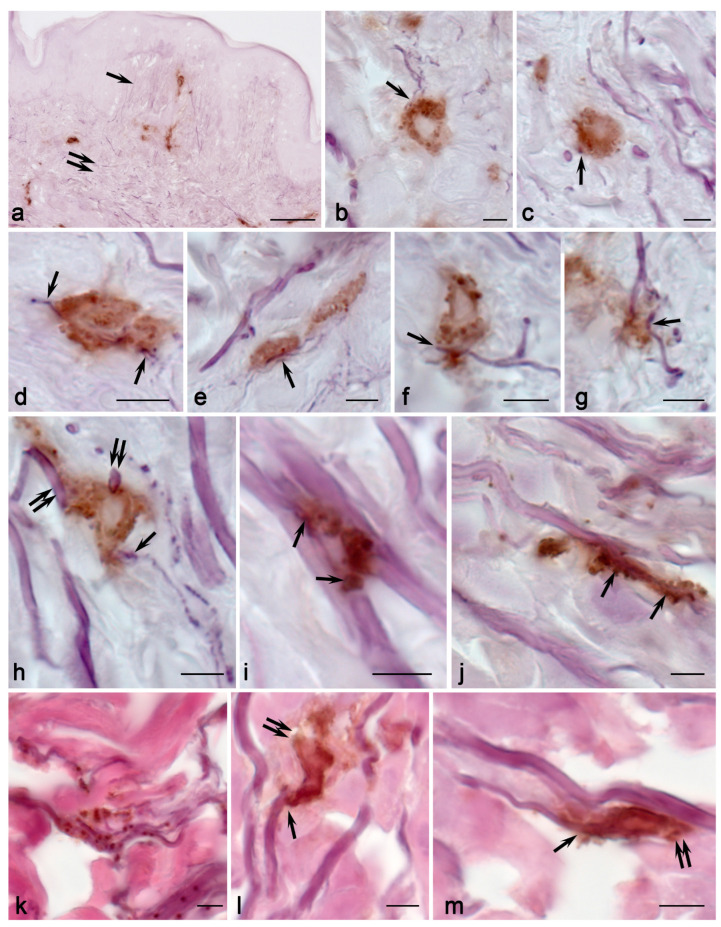
Mast cells in the fibrous landscape of the skin dermis in children without pathology. Technique: (**a**–**j**) Weigert stain combined with tryptase immunohistochemical detection. (**k**–**m**) Weigert–Van Gieson stain combined with immunohistochemical tryptase detection. Notes: (**a**) general view of the elastic network in the papillary (arrowed) and reticular (double arrowed) layers of the skin dermis, with MCs. (**b**,**c**) MC colocalization with thin elastic fibers in the papillary layer of the skin dermis (arrowed). (**h**–**m**) Interaction of MCs with elastic fibers in the reticular dermis. (**h**) MC in contact with several small-caliber (arrowed) and large-caliber (double arrowed) elastic fibers. (**i**,**j**) Tryptase-positive MC granules surrounding thick elastic fibers (arrowed). (**k**) Predominant localization of MC secretory granules in the area of elastic fibers. (**l**,**m**) Target tryptase secretion towards elastic fibers (arrowed) and collagen fibers (double arrowed). Scale: (**a**) 50 µm; the rest—5 µm.

Skin MCs in children without MFS were small cells, ranging in size from 7 to 12–15 μm; their granules were small (up to 0.5–0.7 μm in diameter) and filled with heparin and specific proteases (Figure 3 and Figure 4). MCs were most often accompanied by the dermal vessels located near the basement membrane of endothelial cells (Figure 3f). Smaller MCs were typically localized in the reticular layer of the dermis, while large MCs with well-detected secretory material in granules were located in the reticular layer. MCs were able to interact simultaneously with several fibroblasts and fibrocytes (Figure 3d). Sometimes, MCs in contact with each other formed groups over a considerable distance (up to 50 µm or more in the plane of observation), developing a functional chain that linked specific tissue structures into a single cluster (Figure 3f).

MCs had variability with respect to the cytoplasm’s shape. Elongated MCs with processes spreading over considerable distances (up to 10–30 µm only in the field of view) and more rounded MCs with well-defined granules were both detected (Figure 3a,b). With metachromatic staining, the granules appeared very small, sometimes resembling dust-like stippling (Figure 3h,i). MCs with well-differentiated and filled granules in the cytoplasm were quite rare. The most common arrangement of granules in the cytoplasm was loose, with a prevalent accumulation in the near-membrane area (Figure 3c,k,m).

Interestingly, there were areas in the skin dermis in which separately located granules and thin nuclear-free areas of the MC cytoplasm filled with granules were detected between bundles of collagen fibers (Figure 3g,l). As a rule, they repeated the path of fiber bundles and were located between them (Figure 3g). The size of MCs increased in the deeper layers of the dermis. MCs were colocalized with other dermal cells, including fibroblasts (Figure 3d). MCs were sometimes colocalized with various types of cells in the tissue microenvironment of the skin simultaneously (Figure 3d,f,g). Some MCs were located fairly deep in the skin dermis and came into exclusive contact with the fibers. Metachromatic detection revealed an interaction with the fibrous component of the ECM of the skin dermis, although only bundles of collagen fibers could be observed more objectively (Figure 3). Some MCs had a rather elongated and thin process, and their thickness corresponded to the average size of one secretory granule (Figure 3b).

### 2.2. Skin in Patients with Marfan Syndrome

In MFS, attention should primarily be given to the increased volume of the intraorgan MC population. This was quite evident in both the detection of specific proteases—tryptase, chymase, and CPA3—and CD117 or during metachromatic staining (Figure 1, Supplementary Table S1). The content of tryptase^high^ MCs and CD117-expressing MCs increased especially significantly—almost twice. Notably, an increase in the number of tryptase-positive MCs could reach the one-fifth level of all other cells of the skin dermis; this fact allows considering MCs in MFS as the main regulators of the immune and stromal landscapes of the skin.

**Figure 5:**
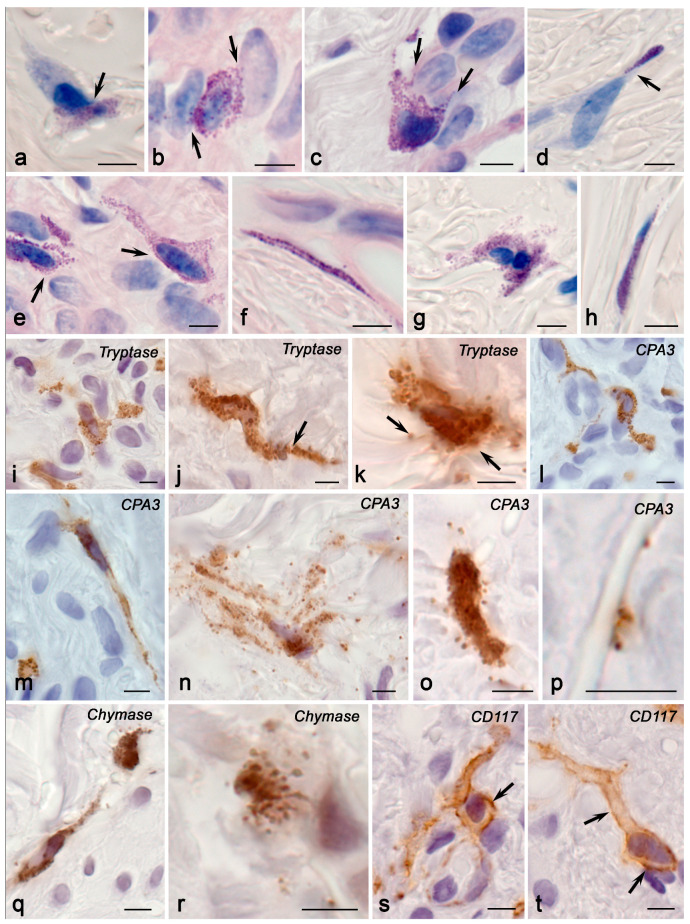
Mast cells in the skin dermis in Marfan syndrome. Technique: (**a**–**h**) staining with Giemsa solution, (**i**–**t**) immunohistochemical staining for tryptase (**i**–**l**), carboxypeptidase A3 (**m**–**p**), chymase (**q**,**r**), and CD117 (**s**,**t**). Notes: (**a**–**e**) options for various MC colocalizations with fibroblasts (arrowed). (**f**) A nuclear-free area in the cytoplasm of an elongated MC. (**g**,**h**) Secretion of heparin-containing granules onto elastic fibers. (**i**) A group of MCs closely localized to fibroblasts. (**j**) Directed distribution of tryptase over long distances in the skin dermis (arrowed). (**k**) Active tryptase secretion onto the elastic fiber (arrowed). (**l**) Group of interacting CPA3^+^ MCs. (**m**) An elongated MC with a predominantly peripheral localization of CPA3 in the cytoplasm. (**n**–**p**) Active targeted secretion of CPA3 onto elastic fibers. MC granules adjacent to elastic fibers. (**q**,**r**) Active chymase degranulation onto fibers and cells of the skin dermis. (**s**,**t**) Predominantly peripheral localization of CD117 in the MC cytoplasm (arrowed). Scale: 5 µm. CPA3—Carboxypeptidase A3.

Analysis of the specific protease profile revealed a predominant Tr^+^Ch^+^CPA3^+^ phenotype, typical of the norm, with low expressions of chymase in terms of MCs (Supplementary Figure S1). Attention was drawn to the frequent detection of narrow nuclear-free sections of the MC cytoplasm; they could only fit two closely packed secretory granules (Figure 5f). Moreover, these granular parts of the MC cytoplasm seemed to be able to pass into each other, although the small thickness of the studied histological section did not allow such functional continuity to be observed in one section (Figure 5c,f,g,j).

**Figure 6:**
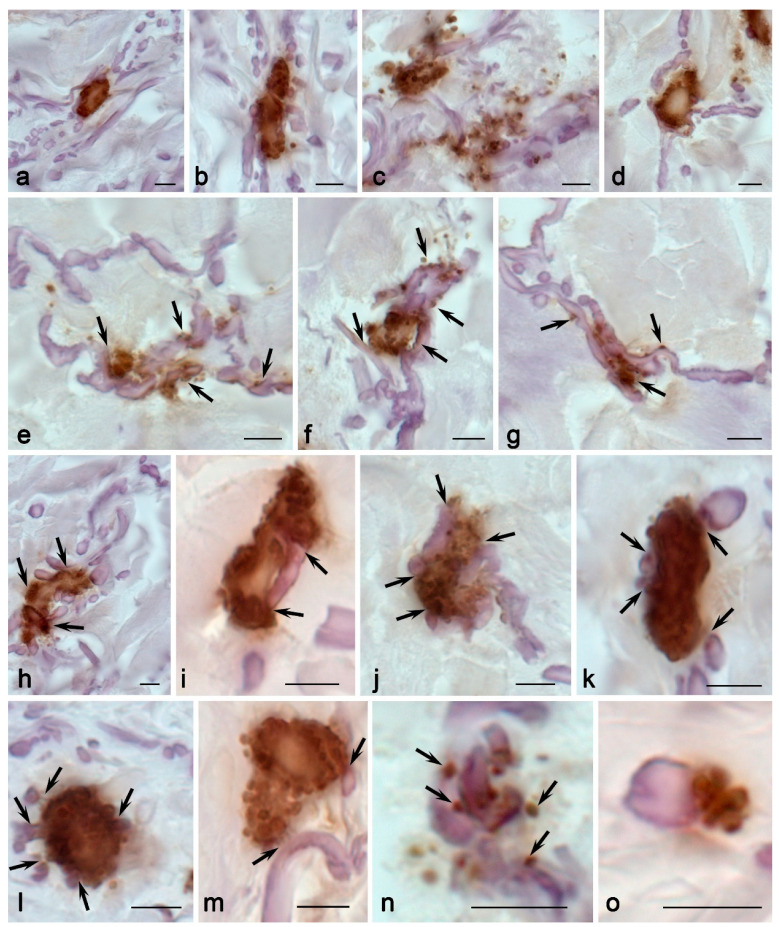
Target secretion of mast cell tryptase to the structural components of the elastic landscape of the skin dermis in Marfan syndrome. Technique: Combined Weigert staining with immunohistochemical detection of MC tryptase. Notes: (**a**,**b**) MC migration to areas with abnormal elastic fibers. (**c**) Intensive degranulation of MC with the development of elastic fiber degradation. (**d**) Spatial localization of MCs between three directions of elastic fibers. (**e**–**g**) Targeted MC degranulation onto selected elastic fibers in the skin dermis (arrowed). (**h**) Accumulation of several MCs in areas of elastic fiber remodeling (arrowed). (**i**–**m**) Targeted tryptase secretion to the selective loci on elastic fibers of various thicknesses (arrowed). (**n**,**o**) Interaction of individual (**n**) or grouped (**o**) secretory granules (**n**) and the external component of elastic fibers from abnormal microfibrils with tryptase accumulation on the surface (arrowed). Scale: 5 µm.

When analyzing the ratio of collagen to elastic fibers in the skin of children with MFS, a noticeable decrease in the content of elastic fibers was detected in only two patients (patient No. 3 and patient No. 6) (Table 1). In all other examined patients, there were no noteworthy quantitative distinctions of this parameter compared to the norm (Table 1). On the other hand, it should be noted that when the integral area of elastic fibers in the skin tended to be close to normal, we revealed a specific topographic distribution of elastic fibers. First and foremost, this is related to the high variability in the elastic fiber’s thickness throughout the entire distance and the diverse spatial landscape of the elastic framework with respect to the fibers’ uneven surface topography (Figure 6).

**Figure 7:**
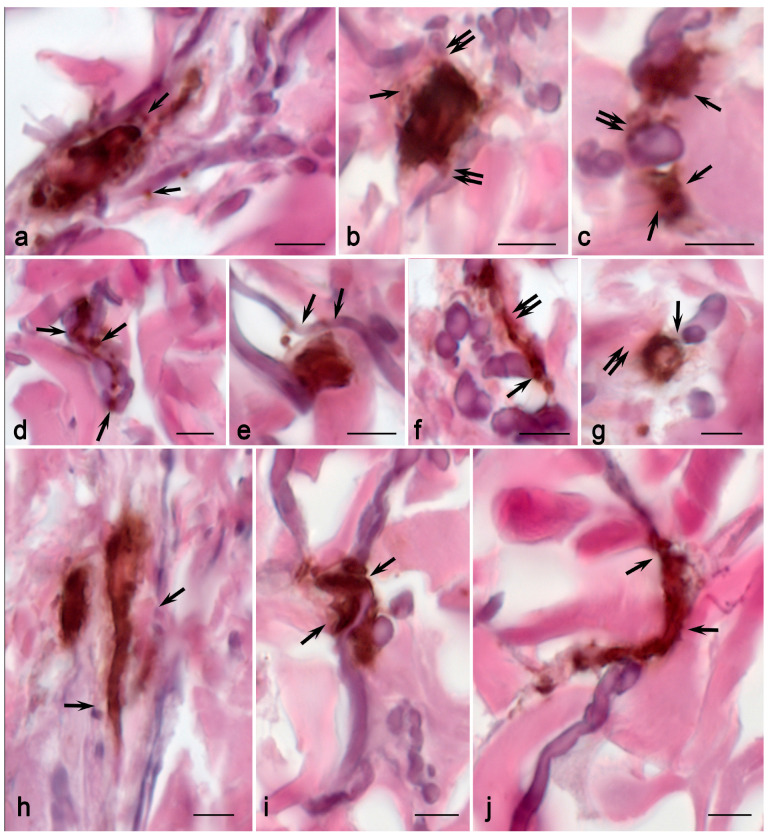
Mast cell mapping in the fibrous landscape of the skin dermis in MFS. Technique: Combined Weigert–Van Gieson staining with immunohistochemical tryptase detection. Notes: (**a**) Simultaneous tryptase secretion onto the collagen and elastic fibers in the skin dermis (arrowed). (**b**) Formation of a pericellular zone of extracellular matrix remodeling (arrowed) and its impact on elastic fibers (double arrowed). (**c**) Active effect of tryptase on the collagen ECM (double arrowed) and extensive areas on the surface of elastic fibers (arrowed). (**d**) Selective localization of autonomous secretory MC granules in the elastic fiber area in the skin dermis (arrowed). (**e**) Selective tryptase secretion onto elastic fibers with a difference in their histochemical properties (arrowed). (**f**,**g**) Active entry of tryptase into areas of contact with collagen (arrowed) and elastic fibers (double arrowed). (**h**) Evident remodeling of the skin dermis by mast cells, with visible signs of elastic fiber destruction (indicated by an arrow). (**i**,**j**) Variants in MC localization in the tensometric areas of the elastic network of the skin dermis (arrowed), with intense tryptase secretion on the surface of the fibers. Scale: 5 µm.

The increased intensity of MC migration to elastic fibers was a crucial sign detected in children with MFS. In all patients, MCs were much more likely to occur in close contact with elastic fibers (Figure 2, Figure 6 and Figure 7; Supplementary Table S2). In addition, it should be underlined that MCs in contact with elastic fibers exhibited high secretory activity (Figure 6). The microphotographs demonstrate how actively protease secretion is accompanied by their targeted localization around elastic fibers. In some instances, the complete encirclement of elastic fibers by secretory material was observed. In particular, secretory CPA3^+^ granules spread along elastic fibers over considerable distances, and they were adjacent to their outer layer—the main locus of defective fibrillin-1 microfibril localization (Figure 6). In some cases, MCs were connected via elastic fibers (Figure 6). There were also elongated MCs that, in fact, acted as conductors between several fibroblasts in the skin dermis, with signs of active tryptase secretion directed towards the fibroblast (Figure 5). Tryptase often accompanies particular fibers for a long time. MC granules that were separately located around the fibrous component were encountered quite frequently (Figure 5). Tryptase could be secreted in large areas of the tissue microenvironment around fibroblasts (Figure 5j).

**Figure 8:**
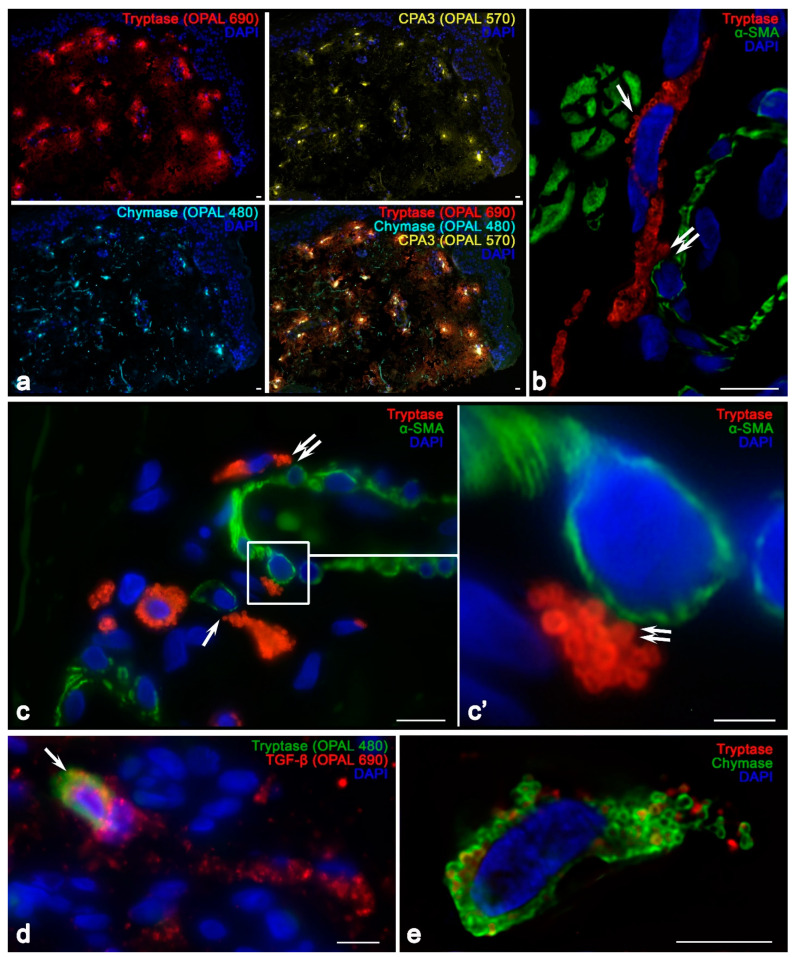
Secretory profile of skin MCs in Marfan syndrome. Technique: Multiplex immunohistochemistry with simultaneous detection of tryptase, chymase, and CPA3 (**a**) and tryptase with αSMA (**b**,**c**) and TGFβ (**d**). Notes: (**a**) MCs in the skin dermis with a predominant Tryptase^+^Chymase^+^CPA3^+^ profile. (**b**,**c**) Colocalization of MCs with smooth myocytes in the microvasculature (double arrowed) and α-SMA-positive cells (arrowed), with active tryptase secretion. (**d**,**e**) High level of TGF-β expression in MCs in the skin dermis (arrowed). Scale: (**c’**) 2 µm; (**e**) 5 µm; the rest—10 µm. CPA3: carboxypeptidase A3. TGF-β: transforming growth factor beta.

In MFS, mast cells more intensively expressed CD117, which was located in the region of the MC plasma membrane, including cytoplasmic outgrowths (Figure 5s,t). This provides evidence of the greater potential of the skin MC population to undergo further differentiation, development, and survival in MFS. In addition, it is necessary to note the increased TGF-β biogenesis in MCs (Figure 8d). In terms of the population structure, MCs with moderate and high contents of TGF β were more common. In terms of histotopography, MCs with a high content of TGF-β were more often located among the fibrous component of the skin dermis.

There was an impression of more actively functioning fibroblasts; part of their cytoplasm became intensively basophilic due to their high synthetic activity (Figure 5a,c). Concurrently, a high level of MC cooperation with fibroblasts could be noted.

It can be asserted that more than half of the MCs were, to some extent, colocalized with fibroblasts, either in direct contact format or at a paracrine distance not exceeding a distance equal to 20–30 µm.

Colocalization with fibroblasts was often accompanied by the active secretion of biologically active substances in the composition of the granules (Figure 5b,e), including tryptase, chymase, and carboxypeptidase A3. Quite frequently, there was an impression that secretory MC granules were transported directly to the fibroblast nuclei (Figure 5j).

It should be noted that isles of the smooth muscle tissue were detected in the skin of children with MFS (Figure 8b). MCs were rarely detected inside smooth muscles as they were mainly located around the muscle layers (Figure 8b). It is evident that this fact caused an increased frequency of MC contact with the SMA-positive cells in the skin dermis in MFS (Figure 2, Supplementary Table S2). MCs were sometimes located in the extracellular matrix between isles of the smooth muscle tissue and the vascular bed (Figure 8b). Moreover, the formation of specific strands of contacting MCs increased the area of contact with SMA-positive elements in the skin. Direct contact of protease-containing secretory MC granules with smooth myocytes in the wall of postcapillary venules was observed (Figure 8c).

MCs formed functional chains over longer distances, connecting more fibroblasts and other dermal cells to each other (Figure 5c). In this case, the chains could accompany elastic fibers (Figure 5g,o). MCs were more often in contact with each other and the fibrous component of the skin dermis, and they sometimes evolved into a fiber (Figure 7h,j). These patterns were much more common than controls.

MCs in children with MFS were in a more active functional state, particularly in terms of secretion mechanisms (Figure 5, Figure 6, Figure 7 and Figure 8). Active degranulation was accompanied by the detachment of small granular formations from larger secretory granules located on the periphery of MCs. In some cases, tryptase-filled nanotubes were formed; they were directed toward targets in the tissue microenvironment, including smooth muscle cells.

## 3. Discussion

As reported, since the skin may be a window into the hereditary diseases of connective tissue [29], the study of its cellular component in MFS is absolutely reasonable. Concurrently, MCs are a critical unit of analysis in hereditary pathologies affecting connective tissues due to their known high potential to regulate local homeostasis. The study of the molecular portrait of MCs can specify how mutations in the FBN1 gene affect changes in the stromal and immune landscapes. In some specified sense, MCs are one of the key sculptors of the connective tissue phenotype, including that in MFS. Furthermore, it can be assumed that in MFS, the revealed patterns of MCs in the skin dermis may be typical of connective tissue in other organs because differences in the macromolecular structure of microfibrils affect all body structures.

The elastic network of the skin consists of bundles of microfibrils (oxytalan fibers) emerging from the dermo-epithelial junction into the dermis, where they interact with thin elastin-containing fibers (elaunin fibers) and form a network of thick-caliber elastic fibers in the deeper layers of the dermis.

Notably, microfibrils in the composition of elastic fibers are considered structural “tensometers”. Their conformational changes during mechanical stretching can indirectly modify the migration, functional activity, and differentiation of cells through integrin receptors [3]. In this aspect, the effect of MCs on the elastic component of the dermis is of particular interest since the targeted secretory activity relative to elastic fibers primarily results in contact of the secretome, including heparin and specific proteases, with fibrillin-1 and microfibrils. This is why the issue of the MC’s potential to compensate for the lost natural interstitial tensinometry of elastic fibers by forming spatial clusters in the connective tissue base of the skin deserves further study. Indeed, the microscopic patterns detected support a more active formation of functional ensembles in MFS than the norm. This process captures wide areas of the skin and synchronizes a larger number of cells from both the immune and stromal landscapes.

In addition, the issue of the MC effect on elastic fibers deserves special attention. The degrading MC effects on elastic fibers are known and have been demonstrated in a few studies with respect to elastinopathy, particularly mid-dermal elastolysis, which is characterized by selective loss of elastic fibers in the middle part of the skin dermis [21]. Close colocalization of degranulating MCs with fragmented elastic fibers has been observed at the ultrastructural level in skin striae atrophicae formation [22]. Notably, elastic fibers were disorganized, had uneven edges and/or signs of elastolysis, and lost the specific peripheral localization of microfilaments [22]. In our studies, we observed patterns of changes in the relief of elastic fibers at the light-optical level.

Nonetheless, the theoretical potential of MCs to degrade elastic fibers is supported by their production of corresponding enzymes, including elastase and cathepsin G [23,24,25,26,27,28].

The active secretion of tryptase, chymase, CPA3, and heparin directly onto elastic fibers, which we observed, may support their direct involvement in the metabolism of the fibrous component of the skin dermis. However, in this regard, a natural question arises regarding the absence of significant changes in the relative ratio of elastic to collagen fibers. We detected a decrease in the relative content of elastic fibers in the skin dermis of only two patients. Moreover, our findings are not consistent with the findings detected in mice under simulated MFS: A significant decrease in the bulk density of elastin and collagen and a thinner skin dermis were found [30]. This may be due to differences in the pathogenesis of MFS between humans and laboratory animals and the severity of genetic pathology manifestations in MFS. In addition, one can argue the MC’s potential to re-synthesize elastic fibers after their degradation into specific structural and molecular units that can subsequently be used to assemble new elastic fibers. This fact could explain the absence of a significant decrease in the integral area of elastic fibers in biopsy skin sections. Previously, we studied the potential of MCs to participate in collagen fibrillogenesis due to direct and indirect effects [18,31].

This speculation can be applied to explain the mechanisms of elastic fiber remodeling since the effect of heparan sulfate (and possibly heparin) on the activity of the fibrillin-1 molecule’s interaction during linear microfibril polymerization is known [3,32,33]. The similarity between heparan sulfate and heparin enhances the potential of MCs in elastic fiber processing. Although MCs and basophils are two major sources of heparin in humans, MCs—according to their prolonged presence and abundance in the skin—are considered the main producers of heparin in the skin [34,35,36]. In this aspect, an increased number of MCs can be considered as a compensatory reaction associated with the need to create additional ECM strength.

On the other hand, the role of MCs in developing fibrotic changes should be taken into account. In particular, as demonstrated, the formation of fibrous niches in skeletal muscles is due to mutations in the FBN1 gene in MFS; this results in an increased accumulation of collagen fibers and fibroblasts [37].

As stated, there is histological evidence of myocardial fibrosis in mice with MFS and an increase in diffuse myocardial fibrosis biomarkers in children and adolescents with MFS [38]. A patient with MFS is reported to develop extensive fibrosis after radiation therapy [39]. In the aorta of patients with MFS, increased expression of contractile protein markers, including α-SMA; accumulation of type I collagen; fragmentation; loss or proliferation of interlaminar elastic fibers; and accumulation of acid mucopolysaccharides have been detected [40,41].

Since MFS represents the pathobiology of fibrillin-1, it should be expected that both the architecture of intercellular interactions and the mechanisms of interaction with the components of the ECM are to be disturbed to a certain extent. Dysfunctional fibrillin-1 fails to form latent TGF-β-binding protein (LTBP) and accumulates TGF-β in the ECM, resulting in an increased release of the latter [42,43]. After release, TGF-β interacts with the TGF-β receptor family, resulting in the regulation of physiological processes such as angiogenesis, cell proliferation, differentiation, apoptosis, and the regeneration and remodeling of the structure and composition of the ECM [44,45,46,47]. As demonstrated, TGF-β has the potential to induce excessive collagen formation and elastic fiber degradation and enhance the proliferation and migration of smooth muscle cells [48,49]. Our findings support increased synthesis of TGF-β in MCs. Furthermore, it should be noted that MCs start to actively deliver the growth factor to tissue targets, thus apparently compensating for its absence in tissue depots (niches). This apparently forces an increased synthesis of TGF-β in MCs; moreover, to improve its functional value without storage, MCs can migrate directly to tissue (cellular) targets, which is followed by growth factor secretion (Figure 8d).

The increased representation of MCs with positive expression of CD117 allows us to conclude that, in MFS, the MC population has a great potential for further differentiation, growth, survival, and high functional activity. The increased number of MCs is a sign of their active participation in the metabolism of the stromal landscape of the skin dermis in MFS. In addition, this fact should be considered as a certain basis for developing distorted immune responses in MFS and a specific response to external and internal stimuli. Active secretory activity, including specific proteases, emphasizes the role of the ECM in developing the fibrous background in response to the decreased strength of the elastic component. In terms of the above, the compensatory functions of MCs turn into pathological ones; this can explain the developing fibrosis in other organs, as demonstrated in a number of studies [37,38,39,40,41].

## 4. Materials and Methods

### 4.1. Case Selection

This study involved skin biomaterial taken during a biopsy from the outer surface of the upper third of the shoulder in patients with MFS who underwent treatment at the Veltischev Research and Clinical Institute for Pediatrics and Pediatric Surgery, Pirogov Russian National Research Medical University, Ministry of Health of the Russian Federation (hereinafter—Veltischev Institute) in 2022–2023. The group of patients with MFS included parents and children: a 45-year-old mother and her 4-year-old daughter (hereinafter referred to as patients No. 1 and No. 2, respectively); an 11-year-old female and a 12-year-old male (hereinafter referred to as patients No. 3 and No. 4, respectively); a 44-year-old mother and her 13-year-old son and 11-year-old daughter (hereinafter referred to as patients No. 5, 6, and 7, respectively). MFS was diagnosed according to the 2010 Ghent criteria [50]. The biopsy was performed from the outer surface of the upper third of the shoulder, which allowed for the minimization of the contribution of organ-specific features of the skin mast cell population from different parts of the human body [51,52]. Normal skin was examined from two children aged 11–12 (hereinafter named norm No. 1 and norm No. 2).

This study was conducted in accordance with the World Medical Association Declaration of Helsinki: Ethical Principles for Medical Research Involving Human Subjects; it was approved by the local ethics committee of the Veltischev Institute, protocol dated 18 November 2022 (Moscow, Russia). Informed consent was obtained from the parents or legal representatives of the children. The samples qualified as redundant clinical specimens that had been de-identified and unlinked from patient information.

### 4.2. Tissue Probe Staining

The tissue probes that were left over from the routine diagnostic procedure were fixed in buffered 4% formaldehyde and routinely embedded in paraffin. The paraffin tissue sections (2.5 µm thick) were deparaffinized with xylene and rehydrated with graded ethanol, according to a standard procedure [53].

### 4.3. Immunohistochemistry and Histochemistry

For the immunohistochemical assay, we subjected deparaffinized sections to antigen retrieval by heating the sections in a steamer with R-UNIVERSAL Epitope Recovery Buffer (Aptum Biologics Ltd., Southampton, SO16 8AD, UK) at 95 °C/30 min. Blocking the endogenous Fc receptors prior to incubation with primary antibodies was omitted, according to our earlier recommendations [54]. After antigen retrieval and, when required, quenching endogenous peroxidase, the sections were immunoreacted with primary antibodies. The list of primary antibodies used in this study is presented in Table 2. The immunohistochemical visualization of bound primary antibodies was performed manually according to the standard protocol [53,55]. For manually performed immunostaining, primary antibodies were applied at concentrations ranging from 1 to 5 µg/mL and incubated overnight at +4 °C. Bound primary antibodies were visualized using secondary antibodies (purchased from Dianova, Hamburg, Germany, and Molecular Probes, Darmstadt, Germany) conjugated with Alexa Fluor-488 or Cy3. The final concentration of secondary antibodies was between 5 and 10 µg/mL PBS. Single and multiple immunofluorescence labeling was performed according to standard protocols [53]. The list of secondary antibodies and other reagents used in this study is presented in Table 3.

Sequential multiplex immunohistochemical staining for the simultaneous detection of tryptase, chymase, carboxypeptidase A3 (CPA3), and CD31 was performed in accordance with Akoya Biosciences (USA) recommendations on the use of OPAL series fluorochromes for the Mantra 2 Quantitative Pathology Imaging System. In addition, when using OPAL series fluorochromes for repeated retrieval, the EZ-Retriever^®^ System, MW015-IR (BioGenex, Fremont, CA, USA), was applied. For the simultaneous detection of tryptase-positive MCs, elastic fibers, and collagen fibers, a combined protocol including standard immunohistochemical tryptase staining and elastic fiber staining was used according to Weigert’s method or a protocol for the simultaneous staining of elastic and collagen fibers according to the combined Weigert–Van Gieson method. The ratio of elastic to collagen fibers was estimated according to the manufacturer’s protocol after simultaneous Weigert–Van Gieson staining.

Histochemical staining with toluidine blue, Giemsa solution, and hematoxylin and eosin was carried out according to the picro-Mallory protocol, Weigert method, and combined Weigert–Van Gieson staining method, and silver impregnation was performed according to the manufacturer’s instructions (Table 3).

### 4.4. Image Acquisition

Stained tissue sections were observed using a ZEISS Axio Imager.Z2 equipped with a Zeiss alpha Plan-Apochromat objective 100×/1.46 Oil DIC M27, a Zeiss Objective Plan-Apochromat 150×/1.35 Glyc DIC Corr M27, and a ZEISS Axiocam 712 color digital microscope camera—a ZEISS Axiocam 712 mono digital microscope camera (Carl Zeiss Vision, Jena, Germany). Captured images were processed with the software programs “Zen 3.0 Light Microscopy Software Package”, “ZEN Module Bundle Intellesis & Analysis for Light Microscopy”, and “ZEN Module Z Stack Hardware” (Carl Zeiss Vision, Jena, Germany) and submitted with the final revision of the manuscript at 300 DPI. In some cases, photomicrographs were obtained with a Nikon D-Eclipse C1 Si confocal microscope based on the Nikon Eclipse 90i. The Mantra 2 Quantitative Pathology Imaging System for multiplex visualization (Akoya Biosciences, USA), based on an Olympus BX43 microscope equipped with a scientific-grade multispectral 12-bit monochrome high-sensitivity CCD camera with a liquid crystal tunable spectral filter, was used to determine the profile of specific mast cell proteases in multiple immunodetection instances of tryptase, chymase, and CPA3 using the OPAL 690, OPAL 480, and OPAL 570 fluorochromes, respectively.

### 4.5. Quantitative Analysis

Quantitative analyses of the studied criteria involved biopsy skin fragments. Planimetric analysis was performed to identify the number of MCs per unit area of skin, the absolute number of MCs and other cells, and the ratio of elastic to collagen fiber areas and specify the profile of specific mast cell proteases via multiplex tryptase, chymase, and CPA3 staining, with the determination of “high”, “moderate”, and “low” expression levels using the QuPath v0.5.1 software product [56]. Stained sections were scanned using the ScanScope CS (Leica Biosystems, Deer Park, IL, USA) and the Mantra 2 Quantitative Pathology Imaging System at ×40 objective, based on an Olympus BX43 microscope, Akoya Biosciences, Marlborough, MA, USA.

### 4.6. Controls

The control incubations were as follows: the omission of primary antibodies or substitution of primary antibodies using the same IgG species (Dianova, Hamburg, Germany) at the same final concentration. The exclusion of either the primary or the secondary antibody from the immunohistochemical reaction and the substitution of primary antibodies with the corresponding IgG at the same final concentration resulted in a lack of immunostaining. The specific and selective staining of different cells with the use of primary antibodies from the same species on the same preparation is, by itself, sufficient control for immunostaining specificity.

## 5. Conclusions

In MFS, skin MCs are actively involved in the connective tissue metabolism of the dermis. An increase in the number of MCs with increased targeted secretion of specific proteases to elastic fibers is one of the compensatory reaction mechanisms that neutralize the violated bioinformatic and spatial functions of abnormal fibrillin-1. The formation of profibrotic MCs in MFS causes parenchymal damage in other organs; this should be taken into account when managing patients according to personalized medicine.

## Figures and Tables

**Table 1:** Profile of collagen and elastic fibers in the skin of patients with Marfan syndrome (technique: simultaneous Weigert–Van Gieson staining).

Patients	Features of the Fibrous Component of the Skin Dermis				
	Total Area of the Fibrous Component of the Skin Dermis (mm^2^)	Collagen Fibers		Elastic Fibers	
		Integral Area (mm^2^)	Relative Content (%)	Integral Area (mm^2^)	Relative Content (%)
Patient No. 1 *	0.66	0.49	74.2	0.17	25.8
Patient No. 2	1.42	1.06	74.65	0.36	25.35
Patient No. 3	2.83	2.51	88.7	0.32	11.3
Patient No. 4	1.83	1.41	77	0.42	23.0
Patient No. 5 **	1.74	1.41	81	0.33	19.0
Patient No. 6	2.13	1.76	82.6	0.37	17.4
Patient No. 7	1.92	1.44	75	0.48	25
The norm No. 1	8.58	6.82	79.5	1.76	20.5
The norm No. 2	12.09	9.17	75.85	2.92	24.15

Notes: *—mother of patient No. 2; **—mother of patients No. 6 and No. 7.

**Table 2:** Primary antibodies used in this study.

Antibodies	Host	Catalogue Nr.	Dilution	Source
Tryptase	Mouse monoclonal	#ab2378	1:3000	AbCam, Cambridge, UK
Carboxypeptidase A3 (CPA3)	Rabbit polyclonal	#ab251696	1:2000	AbCam, UK
Chymase	Mouse monoclonal	#ab2377	1:3000	AbCam, UK
TGF-β	Rabbit monoclonal	#ab215715	1:500	AbCam, UK
Alpha SMA	Mouse monoclonal	#ab7817	1:3000	AbCam, UK

**Table 3:** Secondary antibodies and other reagents.

Antibodies and Other Reagents	Source	Dilution	Label
Goat anti-mouse IgG Ab (#ab97035)	AbCam, UK	1/300	Cy3
Goat anti-rabbit IgG Ab (#ab150077)	AbCam, UK	1/300	Alexa Fluor 488
Secondary antibodies conjugated with horseradish peroxidase (Opal Polymer HRP Ms+Rb (#ARH1001EA))	Akoya Biosciences, Marlborough, MA, USA	ready-to-use	Opal 480 Reagent Pack (#FP1500001KT)
Secondary antibodies conjugated with horseradish peroxidase (Opal Polymer HRP Ms+Rb (#ARH1001EA))	Akoya Biosciences, USA	ready-to-use	Opal 570 Reagent Pack (#FP1488001KT)
Secondary antibodies conjugated with horseradish peroxidase (Opal Polymer HRP Ms+Rb (#ARH1001EA))	Akoya Biosciences, USA	ready-to-use	Opal 690 Reagent Pack (#FP1497001KT)
AmpliStain™ anti-Mouse 1-Step HRP (#AS-M1-HRP)	SDT GmbH, Baesweiler, Germany	ready-to-use	HRP
AmpliStain™ anti-Rabbit 1-Step HRP (#AS-R1-HRP)	SDT GmbH, Baesweiler, Germany	ready-to-use	HRP
4′,6-diamidino-2-phenylindole (DAPI, #D9542-5MG)	Sigma, Hamburg, Germany	5 µg/mL	w/o
VECTASHIELD^®^ Mounting Medium (#H-1000)	Vector Laboratories, Burlingame, CA, USA	ready-to-use	w/o
DAB Peroxidase Substrate Kit (#SK-4100)	Vector Laboratories, Burlingame, CA, USA	ready-to-use	DAB
Mayer’s Hematoxylin (Biovitrum, #05-002)	ErgoProduction LLC, Saint Petersburg, Russia	ready-to-use	w/o
Eosin Y 1% aqueous (Biovitrum, #05-010/S)	ErgoProduction LLC, Russia	ready-to-use	w/o
Giemsa solution (Biovitrum, #21-023)	ErgoProduction LLC, Russia	ready-to-use	w/o
Toluidine blue (Biovitrum, #07-002)	ErgoProduction LLC, Russia	ready-to-use	w/o
Silver impregnation (Biovitrum, #21-026)	ErgoProduction LLC, Russia	ready-to-use	w/o
Picro Mallory trichrome (Biovitrum, #21-036)	ErgoProduction LLC, Russia	ready-to-use	w/o
Weigert for elastic fibers (Biovitrum, #21-030)	ErgoProduction LLC, Russia	ready-to-use	w/o
Weigert–Van Gieson (Biovitrum, #21-020)	ErgoProduction LLC, Russia	ready-to-use	w/o

## Data Availability

Study data are available from the corresponding author upon reasonable request.

## Supplementary Materials

The supporting information can be downloaded at https://www.mdpi.com/article/10.3390/ijms25179191/s1.

## Abbreviations

MFS	Marfan syndrome
MCs	Mast cells
ECM	Extracellular matrix
TGF-β	Transforming growth factor-beta
CPA3	Carboxypeptidase A3
α-SMA	Alpha smooth muscle actin
VEGF	Vascular endothelial growth factor
IL	Interleukins
bFGF	Basic fibroblast growth factor
NGF	Nerve growth factor
PDGF	Platelet-derived growth factor
