# Supplementary material for: The Contribution of Mast Cells to the Regulation of Elastic Fiber Tensometry in the Skin Dermis of Children with Marfan Syndrome
Source: Int J Mol Sci. 2024 Aug 24;25(17):9191. doi: 10.3390/ijms25179191 (PMC11394836; doi:10.3390/ijms25179191)
Supplement: Supplementary file 1 [file ijms-25-09191-s001.zip › ijms-3128910-supplementary.pdf]

Table S1. Mast cell content in the skin of patients with Marfan syndrome.

Patients	Features of the studied sample of the skin dermis				
	Areas (mm^2)	Total number of cells (absolute content)	Total number of mast cells (absolute content)	Relative content of mast cells (%)	Number of mast cells (per mm^2)
CPA3^{high} MCs					
Patient №1*	3.86	2025	414	20.4	107.2
Patient №2	2.4	2110	347	16.4	144.5
Patient №3	4.1	1807	216	11.95	52.8
Patient №4	2.99	1582	288	18.2	96.3
Patient №5**	2.66	1225	197	16.8	74.1
Patient №6	3.4	1998	299	14.96	88
Patient №7	2.44	2335	215	9.20	88.1
The norm №1	13.1	9892	1080	10.9	82.4
The norm №2	18.49	12970	1298	10	70.2
Tryptase^{high} MCs					
Patient №1*	3.84	2096	464	22.1	120.8
Patient №2	2.2	2367	464	19.6	210.9
Patient №3	4.2	1606	293	18.2	69.7
Patient №4	3.07	2085	476	22.8	155
Patient №5**	3.64	2467	365	14.8	100.3
Patient №6	3.89	3618	559	15.5	143.7
Patient №7	2.82	4100	375	9.15	133
The norm №1	12.6	9622	1123	11.7	89.1
The norm №2	17.8	13004	1203	9.25	67.58
Chymase^{high} MCs					
Patient №1*	3.0	1796	174	9.68	58
Patient №2	2.4	1924	265	13.7	110.4
Patient №3	3.3	1832	228	12.8	69
Patient №4 (	3.1	1921	159	8.27	51.3
Patient №5**	3.7	1422	215	15.1	58.1
Patient №6	4.2	1863	301	16.5	71.7
Patient №7	2.7	2526	250	9.89	92.6
The norm №1	11.8	9554	588	6.14	49.8
The norm №2	17.4	15350	1007	6.5	57.9
CD117^{high} MCs					
Patient №1*	3.5	1618	291	15.2	82.9
Patient №2	2.2	2043	354	17.3	160.9
Patient №3	3.6	1226	111	9.0	30.8
Patient №4 (	2.3	1398	295	21.1	128.2
Patient №5**	3.8	3006	375	12.47	98.7
Patient №6	4.2	2836	418	14.7	99.5
Patient №7	3	3241	326	10.1	107.7
The norm №1	11	5115	367	7.2	33.4
The norm №2	12	7428	413	5.6	34.4

Notes: *- mother of Patient No.2, ** - mother of Patients No.6 and No.7.

Table S2. Histotopography of tryptase-positive mast cells in the skin dermis of patients with Marfan syndrome

Patients	Frequency of colocalization (%)			
	Fibrous component of the extracellular matrix of the skin dermis[1]		SMA+ cells[2]	Fibroblasts[3]
	Elastic fibers[1]	Collagen fibers[1]		
Patient №1*	31.2	64.5	2.1	2.2
Patient №2	28.4	63.8	5.6	2.2
Patient №3	36.2	60.6	2.1	1.1
Patient №4	34.7	62.5	2.5	0.3
Patient №5**	36.5	62.1	1.4	1.8
Patient №6	29.90	62.0	5.5	2.6
Patient №7	32.1	63.3	3.1	1.5
The norm №1	12.1	84.4	1.2	2.3
The norm №2	19.8	77.3	1.4	1.5

Notes: *- mother of patient №2, ** - mother of patients №№ 6, 7. Staining technique: [1] multiplex detection of tryptase, elastic, and collagen fibers; [2] multiplex immunohistochemical detection of tryptase and alpha-smooth muscle actin (α-SMA); [3] Giemsa staining.

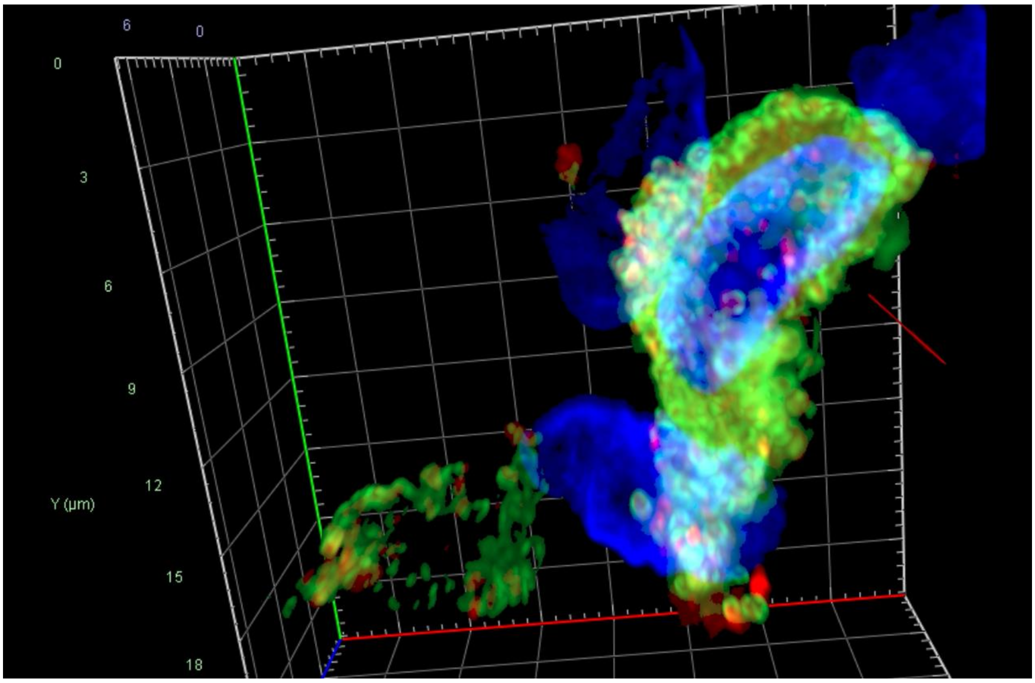

Figure S1. Spatial phenotyping of tryptase (green) and chymase (red) in mast cells in Marfan syndrome (3D model).
